# Recalcitrant Flexor Hallucis Longus Dysfunction: A Case Study Demonstrating the Successful Application of an Adaptable Rehabilitation Program With a Two-Year Follow-Up

**DOI:** 10.7759/cureus.14326

**Published:** 2021-04-06

**Authors:** David P Newman, Kimberley C Holkup, Aimee N Jacobs, Andrew C Gallo

**Affiliations:** 1 Pain Management-Physiotherapy, Tripler Army Medical Center, Honolulu, USA; 2 Physical Therapy, Tripler Army Medical Center, Honolulu, USA; 3 Radiology, Brook Army Medical Center, San Antonio, USA

**Keywords:** flexor hallucis longus dysfunction

## Abstract

Flexor hallucis longus (FHL) dysfunction is a condition experienced primarily by athletes, including ballet dancers and runners. Accurate diagnosis and definitive treatment at the initial evaluation can often be difficult given the number of foot and ankle pathologies that share similar signs and symptoms. The evaluation process tends to be a diagnosis of inclusion rather than a specific pathology with an accepted rehabilitation plan. For example, patients with medial arch pain may undergo an extended rehabilitation period with an evolving differential diagnosis requiring several treatment modifications. A more appropriate rehabilitation paradigm should adapt to the potential changes in patient symptoms and presentation, addressing functional impairments as they arise. This case study describes the successful management of a patient with chronic FHL dysfunction, leveraging a flexible, multimodal treatment approach to address the evolving functional impairments rather than focusing on a single, discrete diagnosis. At a two-year follow-up, the patient remains pain-free.

## Introduction

Foot and ankle injuries are common in the general population with a prevalence ranging from 10% to 24% per year [1,2]. A relatively small subset of these injuries is found in athletes, especially ballet dancers and runners, involving the FHL [3-5]. The challenge in evaluating and managing these injuries is that signs and symptoms of FHL injury overlap with other foot and ankle injuries. The differential for medial arch pain includes, but is not limited to, FHL tendinopathy or tenosynovitis, flexor digitorum longus (FDL) tendinopathy, tarsal tunnel syndrome, and plantar fasciitis [5,6].

The FHL originates from the lower two-thirds of the posterior fibula and interosseous membrane and runs to its distal insertion at the first distal phalanx [7]. The FHL tendon is vulnerable to compression or friction at several locations: first, by an enlarged os trigonum, especially implicated in ballet poses as the FHL tendon is compressed against the ossicle while the foot is in plantar flexion; second, distally at the sesamoid bones of the first hallux; finally, an injury may occur at its intersection in the plantar mid-foot with the crossing FDL tendon, also known as the “knot of Henry” [8,9]. At this location, the tendon sheaths of the FHL and FDL typically communicate, allowing fluid or inflammation to spread from one sheath to the other. Magnetic resonance image (MRI) findings of FHL tenosynovitis at the knot of Henry will often demonstrate peritendinous fluid around both the sheaths of the FHL and FDL [10].

The FHL functions to supinate the subtalar joint, stabilize the medial longitudinal arch, flex the great toe, and stabilize the first metatarsal head. Due to the FHL's importance in generating power during gait, individuals who participate in activities requiring repetitive push-off or plantar flexion can injure the FHL due to overuse or overloading. FHL tenosynovitis, defined as irritation between the tendon and the synovial sheath, is the most common of these injuries and occurs where the FHL passes the posterior aspect of the talus and becomes compressed with repeated stress [3]. Patients with FHL tenosynovitis report pain posterior and inferior to the medial malleolus which is aggravated with plantar flexion.

Due to the anatomy and biomechanics of the FHL, it is rarely injured in isolation. The FHL crosses several other structures in the leg, ankle, and foot. FHL pathology is better described as FHL dysfunction rather than discrete diagnoses. FHL dysfunction has been defined as the primary presence of FHL tenosynovitis combined with the overlapping presentations of FHL tendinopathy, plantar fasciitis, and tarsal tunnel syndrome [5]. Without addressing these other structures, FHL pathology often cannot be managed effectively, resulting in persistent pain and functional limitations [3,6].

The purpose of this case report is to describe the application of an adaptive rehabilitation program, including joint mobilization and manipulation, tissue mobilization, exercises to optimize load upon the FHL and FDL, and short periods of orthosis use, for a patient with recalcitrant medial arch pain.

## Case presentation

A 23-year-old athletic male presented with a 14-month history of left foot medial arch pain. The initial onset of pain was attributed to a six-mile hike carrying 75 pounds of weight on his back as part of an elite training program. The pain was initially produced with walking, jogging, impact activities, and with manual resistance applied to the foot during plantar flexion and inversion of the ankle. The pain persisted for five weeks despite conservative treatment to include an initial course of physical therapy, at which point the patient was issued an orthopedic walker boot by the primary care physician (PCP). The goal was to allow the patient to remain functional while reducing potential re-injury or prolonging the inflammatory process. This treatment approach was similar to that in a clinical study described by Michelson and Dunn [3]. In this approach, immobilization is utilized when stretching of the FHL is too painful or when pain persists despite an adequate course of stretching.

**Figure 1:**
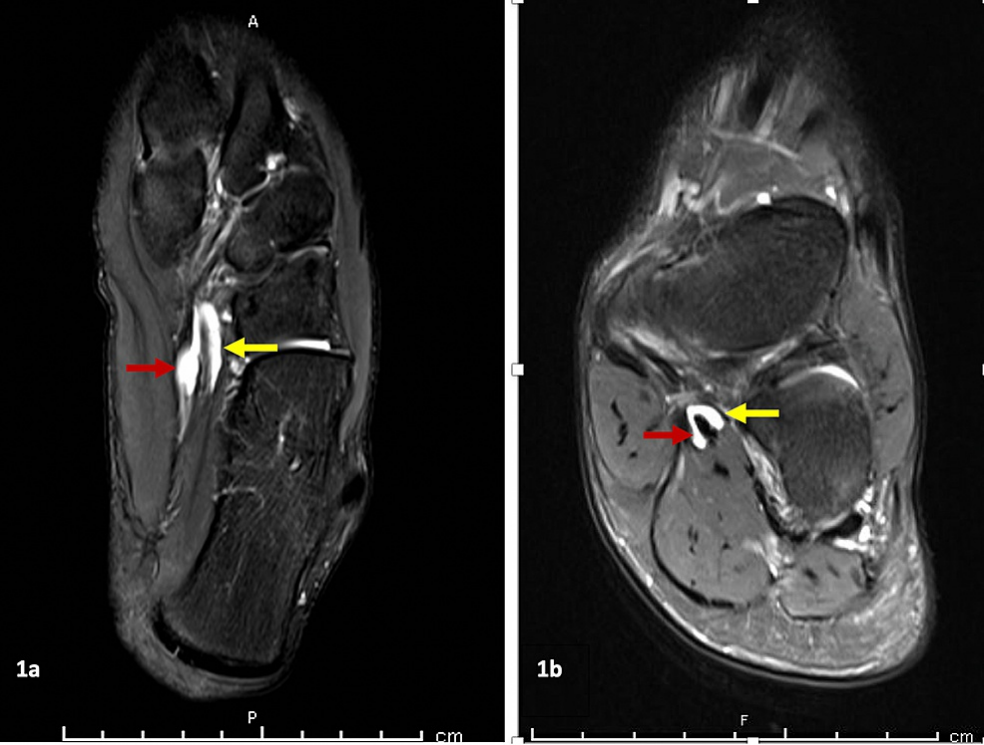
Axial (1a) and Coronal (1b) T2-weighted MRI of Peritendinous Fluid around the FHL (yellow arrow) and the FDL (red arrow) Sheaths. FHL = flexor hallicus longus FDL = flexor digitorum longus

After two weeks, the pain persisted with weight-bearing and he was prescribed crutches by his PCP. The patient was referred for a second course of physical therapy two months post-injury. Under physical therapy guidance, the patient underwent a six-week rehabilitation program. The initial plan included reducing the use of crutches and boot along with instruction in a home exercise program consisting of gastrocnemius and soleus stretching and ankle strengthening. Clinic-based treatment was performed twice a week for three weeks to address strength, flexibility, and proprioception deficits. During this period, an MRI was ordered. The only pertinent finding was tenosynovitis of the FHL and FDL in the axial plane (Figure 1a) and in the coronal plane (Figure 1b). At reassessment six weeks later, a progressive walk to jog at his own pace plan was added to his home program.

After six months of persistent pain, the patient was referred to a podiatrist. Treatment options offered included 4 mg of methylprednisolone three times a day for six days, which did not provide significant relief. Reassessments were performed by the podiatrist two and four months after the initial evaluation. At the first follow-up visit, the patient’s pain area had expanded to include the heel. Plantar fasciitis was added to the diagnosis and custom orthotic shoe insoles were fabricated. The patient did not have morning pain along the heel, a classic symptom of plantar fasciitis [11].

At the next visit two months later, posterior tibialis tendon tenderness was appreciated in addition to chronic medial arch pain, and the differential evolved to include posterior tibialis tendonitis. The patient was referred for the third time to physical therapy for the additional posterior tibialis tendonitis diagnosis; however, given the chronicity of symptoms and lack of response to previous courses of physical therapy, the patient was redirected to physical medicine and rehabilitation (PM&R) specialist. A repeat MRI demonstrated the same FHL and FDL tenosynovitis as compared to the previous one; however, there were no MRI findings consistent with plantar fasciitis. The PM&R specialist considered performing an ultrasound-guided steroid injection into the affected FHL tendon sheath, but knowing that this would not alleviate the underlying biomechanical dysfunction, the patient was subsequently referred to physical therapy within the Interdisciplinary Pain Management Clinic (IPMC) for evaluation and management. Upon initial presentation, the patient’s goals were diagnostic clarity and to pass work-based physical fitness tests.

Physical examination

Physical evaluation at the IPMC revealed that the patient’s pain was localized to the plantar surface of the left medial arch, the abductor hallucis muscle below the navicular bone, and along the flexor digitorum, posterior tibialis, and flexor hallucis tendons superior to the tarsal tunnel. The pain was reported as a 5/10 on a visual analog scale where 0 indicated no pain and 10 being the worst pain imaginable.

The patient reported pain increased to a 10/10 with running. In standing, the patient was apprehensive to fully bear weight on the involved side; consequently, he would shift his weight to the unaffected foot. An antalgic gait was appreciated with intense pain at the terminal stance. Ankle range of motion was within normal limits except active dorsiflexion, which was limited to 5 degrees due to tightness of the gastrocnemius/soleus complex. Passive dorsiflexion was within normal limits. Manual muscle testing revealed pain provocation with the contraction of the FDL and FHL muscles. Joint mobility testing was performed. Translation of the first, second, and third metatarsals was painful. Loading of the first metatarsal reproduced pain in patients with FHL tenosynovitis by mimicking stress placed upon the first ray as the heel rises prior to the foot leaving the ground [3]. The distraction of the talocrural joint revealed hypomobility and pain provocation at the medial arch.

Diagnosis/prognosis

Differential diagnosis specific to this patient’s symptoms upon initial evaluation to the IPMC were extensive and included posterior impingement syndrome, plantar fasciitis, os trigonum, intersection syndrome at the master knot of Henry, tibialis posterior tendinopathy, FDL and FHL tendinopathy, and tarsal tunnel syndrome. Due to the lack of morning pain, the lack of paresthesias, and no accessory bone on imaging, plantar fasciitis, tarsal tunnel syndrome, and os trigonum, respectively, were low on the differential. An MRI did show tenosynovitis of the FHL and FDL in this patient. Given the chronicity of symptoms and his signs and symptoms, the working diagnosis was consistent with a tendinopathy /dysfunction of the FHL, FDL, or both.

The prognosis for full resolution of symptoms was moderate to poor given the chronicity of symptoms, the poor response to adequate trials of physical therapy, lack of response to anti-inflammatories and immobilization, and the patient’s expectations to be pain-free. However, the patient was very motivated and wished to proceed with treatment.

Intervention

The plan of care included manual therapy, stretching, strengthening, instrument-assisted soft tissue mobilization (IASTM), and activity modification (Table 1). The rehabilitation program was designed to incorporate serial assessments, clinic-based osteopathic manipulation technique (OMT) and IASTM, and a self-management program between visits that were to occur every one to two weeks based on his response to each treatment.

**Table 1:** Overview of Interventions Applied and Patient Response at Key Assessment Periods (i.e. Periods When the Working Diagnosis Evolved). FDL = flexor digitorum longus OMT = osteopathic manipulation technique FHL = flexor hallucis longus

	Initial Evaluation	Six-Week Re-evaluation (Visit 4)	Sixteen-Week Re-evaluation (Visit 7)	Thirty-one Week Re-evaluation (Visit 11)
Pain Presentation	Plantar surface of medial arch pain. Abductor hallucis longus muscle belly pain. Pain along the tendons just superior to the tarsal tunnel.	Plantar fascia pain with passive dorsiflexion of the toes. Pain along the FDL tendon when contracted against resistance.	Pain along the navicular bone.	Pain along the tarsal tunnel and transient paresthesias with running over two miles. Positive Tinel’s sign over tarsal tunnel.
Pain Level	5/10 at rest 10/10 with jogging.	1/10 in standing. 5/10 prolonged walking.	4/10 in standing.	0/10 at rest or in standing. 4/10 at jogging 2 miles.
Intervention(s)	Talocrural joint OMT. Manual deep tissue mobilization to the FDL and FHL tendons above the medial extensor retinaculum. Home exercise program.	Plantar fascia orthosis (PFO) fabricated to be worn consistently for one week	Navicular bone mobilization. PFO reapplied for one week	Active cupping to the tarsal tunnel by applying a suction cup over the tarsal tunnel and the patient performs active dorsiflexion and plantar flexion.
Response to Intervention(s)	Immediate increase in pain with treatment. Less pain with ambulation after treatment.	No plantar fascia pain after one week.	Pain decreased to 1/10 after one week of PFO use.	Negative Tinel’s sign.

**Figure 2:**
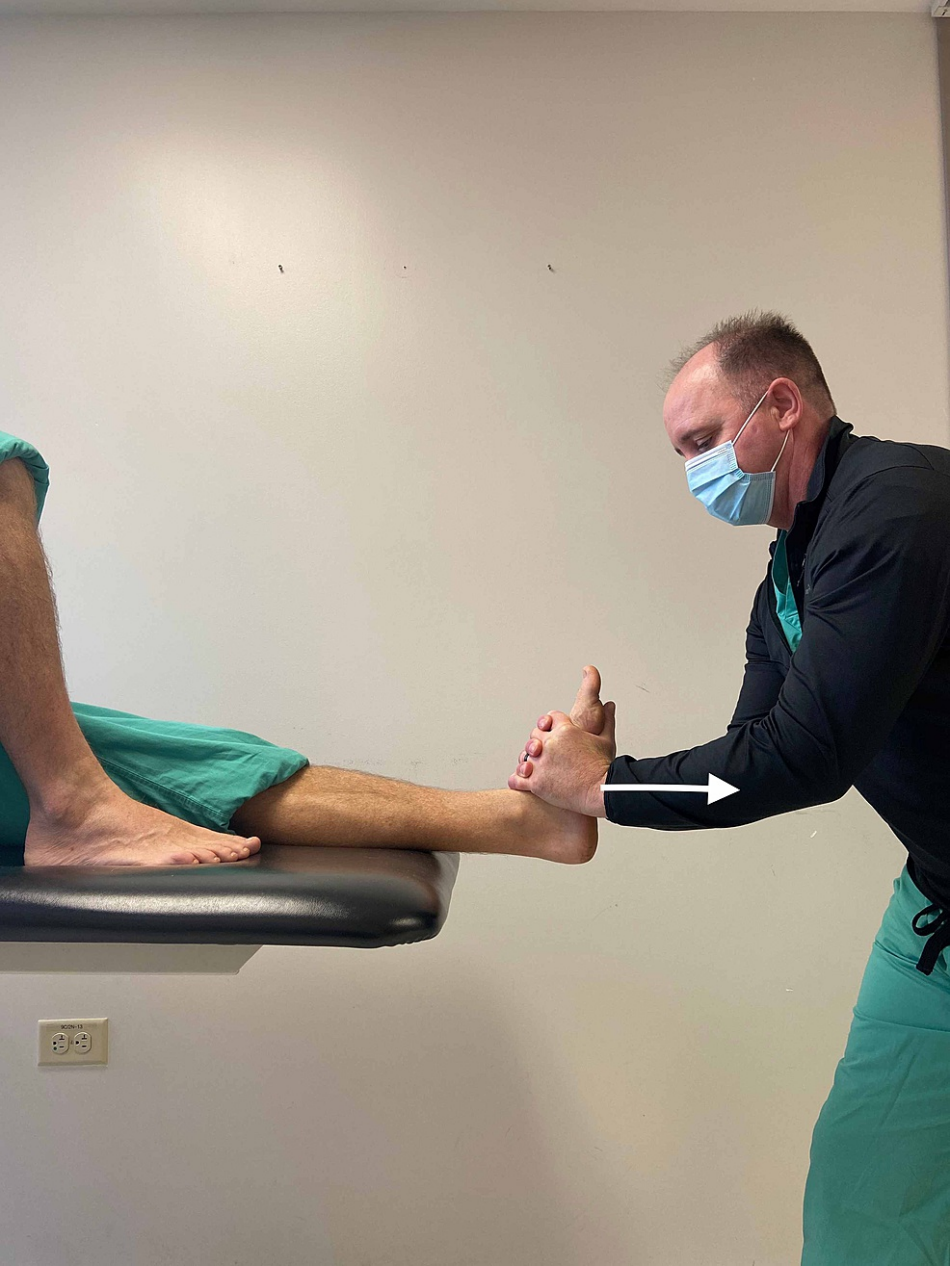
Osteopathic Manipulation Technique – Talocrural Distraction (white arrow). The osteopathic manipulation technique involved grasping the patient’s foot below the tibiofibular joint, applying long axis distraction until a barrier is felt, and then performing a thrust in a caudal direction. *(Photograph: Newman, DP. Osteopathic Manipulation Technique – Talocrural Distraction. Reproduced by permission of the author: 2021.)*

The patient was treated during the initial evaluation with OMT directed at the talocrural joint described by Whitman et al. (Figure 2) and deep tissue mobilization [12]. OMT has been shown to be effective in redistributing load in the foot in patients following ankle inversion injuries and may correct faulty subtalar motion in patients with FHL stenosing tenosynovitis [13,14]. Another potential benefit of this treatment method includes an analgesic effect [15]. Manual deep tissue mobilization was applied to the FDL and FHL muscles and tendons superior to the inferior extensor retinaculum for five minutes. This is the same area that these tendons are accessed during a posteromedial approach for tenolysis [16]. Both techniques provoked an immediate increase in pain. However, after treatment, the patient reported significantly less pain during ambulation. The patient was instructed to perform the tissue mobilization technique daily utilizing a racquetball. The patient would lie on his right side placing the medial aspect of his lower third of the left lower leg on top of the racquetball. He then would slide his leg up so that the racquetball rolled towards the medial ankle and back for a period of two to five minutes. Other home exercises prescribed included intrinsic muscles strengthening by scrunching a towel with his toes to offset the force of the extrinsic muscles and gastrocnemius and soleus stretching for a period of two weeks.

Upon follow-up (two weeks after the initial evaluation), the patient reported a short-term reduction in pain, but pain did return to his baseline pain level of 5/10 due to participating in a work-related exercise event. Tenderness to palpation was appreciated along the medial arch and plantar surfaces, as well as the FDL and FHL at the tarsal tunnel. The initial treatment of OMT to the talocrural joint was repeated. IASTM with an Arthrostim® (Salem, Oregon) was applied to the FHL, FDL, and heel cord. The patient was instructed to focus his self-tissue mobilization along the FDL and FHL tendons between the muscle belly and the inferior extensor retinaculum for another two-week period.

**Figure 3:**
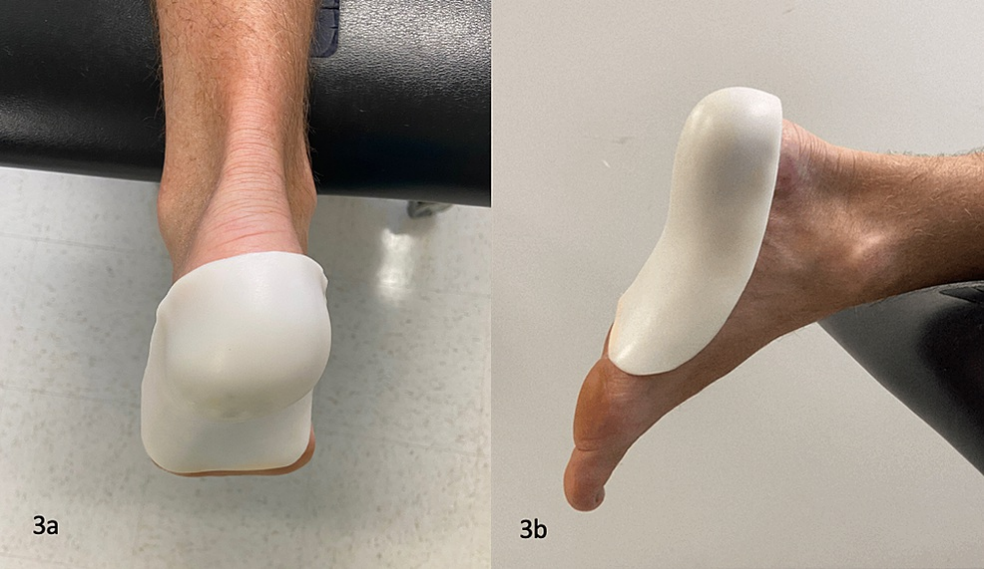
Plantar Fascia Orthosis (a & b). Photograph: Newman, DP. Plantar Fascia Orthosis. Reproduced by permission of the author: 2021.

By the third visit (four weeks after the initial evaluation), the patient’s pain level ranged from a 1/10 to a 5/10. No pain was appreciated with palpation of or contraction of the FHL. The FDL continued to be painful during testing. The previous treatment was repeated with a reduction in pain. The patient was reassessed two weeks later, with reported pain continuing to range from 1/10 with standing to 5/10 with prolonged walking. Notably, less pain was reproduced with the contraction of the FDL. There was pain along the plantar fascia with passive dorsiflexion of the toes, which is indicative of plantar fasciitis [11]. While there was no arch pain in the morning, it was prudent to treat these symptoms as presumptive plantar fasciitis. To unload the plantar fascia while weight-bearing, a custom fabricated plantar fascia orthosis (PFO) was applied [17]. Figure 3a shows the PFO applied in the frontal plane while Figure 3b shows the PFO applied in the sagittal plane. The patient wore this orthotic consistently in his shoes and boots. After one week, the patient’s pain level was 0/10. The physical exam was benign and jogging on a treadmill did not produce pain with push-off. The PFO was discontinued and the patient started a walk to jog progression.

At the sixth visit (two months after the initial evaluation), the patient reported no pain after running one mile on three occasions over the previous week. The patient was instructed to slowly increase his jogging distance with the goal of jogging two miles, a requirement to pass a physical fitness test. The patient was instructed to follow up if he experienced any recurrence in pain.

Two months later (four months after the initial evaluation), the patient re-injured his foot after running down a steep decline involving 1000 steps. The examination revealed pain along the navicular bone with pressure applied from a superior to inferior direction and pain along the plantar fascia. Navicular mobility was addressed with OMT, which immediate reduction in pain while weight bearing from a 4/10 to a 2/10. The PFO was reapplied for one week, thereby reducing the plantar pain to a 1/10. The patient followed up two weeks later for a video-based running assessment. The patient was instructed to improve his arm swing in the sagittal plane and try a mid-foot running technique.

The patient enjoyed another two-month period of pain relief, but he returned with a new complaint of pain along the posterior tibialis tendon. It was presumed that switching to the mid-foot running technique may have resulted in these symptoms. IASTM to the posterior tibialis muscle from the origin to the tendon above the inferior extensor retinaculum resulted in no pain after treatment. One month later, the patient was reassessed (11th visit). The patient reported pain and transient tingling along the tarsal tunnel after jogging greater than two miles. Significant swelling of the FHL sheath can compress the adjacent posterior tibial nerve [6]. A Tinel’s sign was present with reproduction of the paresthesia. The Tinel’s sign has a sensitivity of 0.58 in assessing tarsal tunnel syndrome [5].

**Figure 4:**
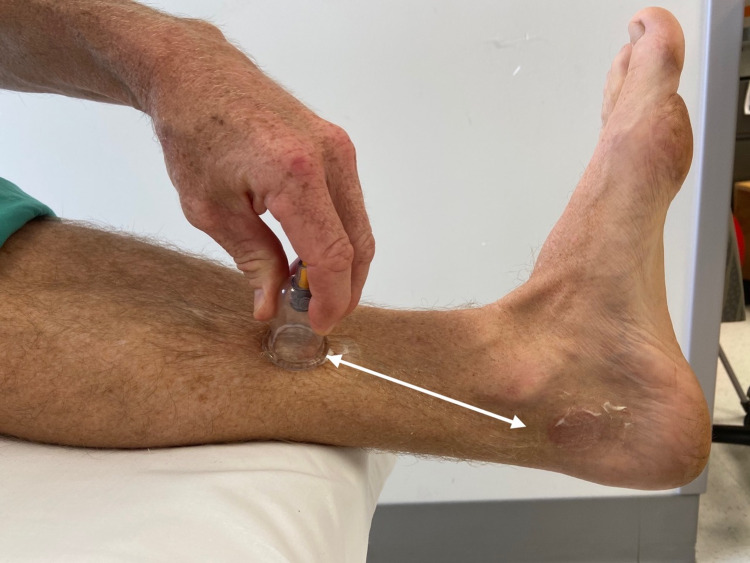
Directional Cupping Technique. A 3.5-cm diameter vacuum suction cup was moved inferiorly and superiorly along the length of the posterior tibialis for 30 seconds as outlined by the white arrow. *(Photograph: Newman, DP.  Directional Cupping Technique. Reproduced by permission of the author: 2021.)*

**Figure 5:**
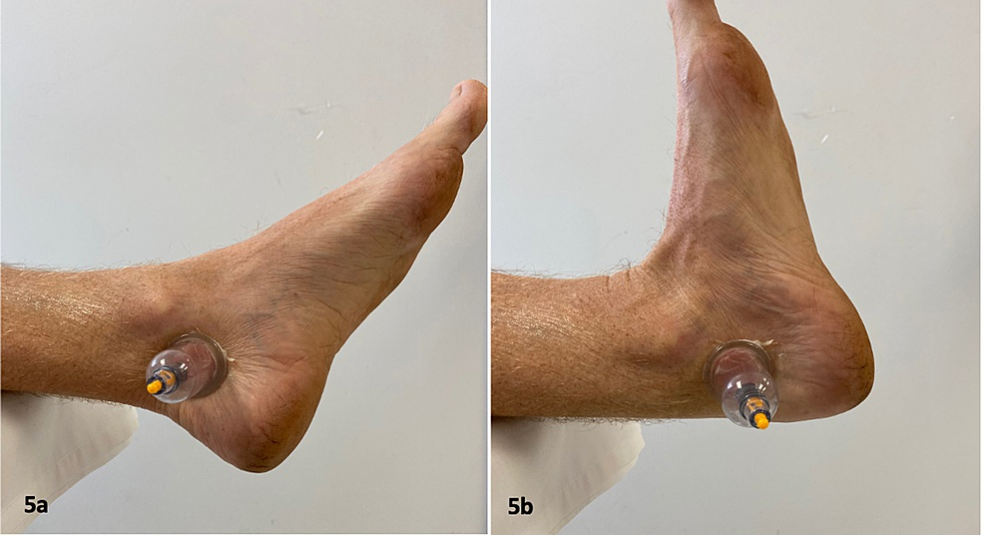
Active Cupping Technique (a & b). A 3.5-cm vacuum suction cup was applied over the tarsal tunnel and the patient was asked to continuously dorsiflex and plantarflex the unweighted ankle for 10 minutes. *(Photograph: Newman, DP. Active Cupping Technique. Reproduced by permission of the author: 2021.)*

IASTM was applied using two cupping techniques. Active lubricating the skin with lotion, a 3.5 cm diameter vacuum suction cup (ZangZhu, China) was used for directional cupping (Figure 4) along the course of the posterior tibialis muscle and active cupping over the tarsal tunnel as the patient plantar flexes (Figure 5a) and dorsiflexes the foot (Figure 5b). These procedures were performed during two consecutive visits. After the second visit, the patient was able to jog greater than two miles without symptoms.

The patient’s primary goal was to complete a work-related physical fitness test. The test took place five months following his last clinic visit, during which he successfully completed the test, running two miles in 14 minutes. The next functional goal was to pass a new, more physically demanding, fitness test, which was scheduled six months later. The patient reported one occurrence of pain along the FHL while sprinting. These symptoms resolved by hill running as an alternative option to sprinting and buddy taping the first and second digits. The patient successfully completed the test without pain. Two years after discharge from the IPMC, the patient was contacted telephonically, at which point he reported no recurrence in pain.

## Discussion

Current evidence describing the efficacy of conservative management versus surgical treatment of FHL dysfunction is equivocal. Several studies report poor outcomes from conservative treatment while others demonstrate good to excellent results in pain reduction and functional improvement [3,5,6]. In a clinical study involving 81 patients diagnosed with FHL tenosynovitis seen in a five-year period, 71% patients underwent conservative management while 28% opted for surgery. Of those electing conservative management (i.e. FHL stretching with or without immobilization and nonsteroidal anti-inflammatory medications), 64% reported improvement while all patients undergoing surgery improved [3]. FHL tendoscopy is indicated for chronic tenosynovitis and stenosing tenosynovitis [16,18]. After a comprehensive review of current literature on tendoscopy of the foot and ankle, the authors felt there is weak evidence supporting tendoscopy of the FHL and insufficient evidence to recommend tendoscopy of posterior tibialis and FDL over conservative management alone [19].

This case report describes the successful conservative management of a patient with FHL dysfunction via a multi-modal rehabilitation program. The program was designed to correct biomechanical faults at the talocrural and talocalcaneonavicular joints, improve intrinsic flexor strengthening, optimize extrinsic flexor muscle and tendon mobility, and lengthen the FHL and gastrocnemius / soles complex. The key to success is a flexible therapeutic approach that adapts to changing signs and symptoms with evolving differential.

Patients with a working diagnosis of FHL dysfunction can present with pain anywhere along the structure’s length from the posterior border of the fibula and interosseous membrane, along the plantar surface of the heel, and extending to the great toe [3,5]. The patient may present with a cluster of symptoms consisting of localized pain and swelling, alteration of ankle and foot mechanics, and structural changes to the tendon [3]. Given the length of the FHL and surrounding structures, the differential can be extensive or a diagnosis of inclusion after failure of conservative management of common posterior foot pathologies such as tarsal tunnel syndrome, posterior tibialis tendonitis, and plantar fasciitis [3].

The evolving differential encountered by the podiatrist was mirrored during the IPMC’s rehabilitation program. First, the patient’s pain pattern, imaging, and physical examination results were suggestive of tenosynovitis of the FHL and FDL tendons. The patient then reported pain along the plantar surface with activity. This would suggest metatarsalgia or potential plantar fasciitis without the classic morning pain symptoms [6]. Finally, the patient experienced pain along the posterior tibialis tendon at the tarsal tunnel suggestive of posterior tibialis tendonitis or tarsal tunnel syndrome. Such a cluster of symptoms may represent several isolated injuries or a spectrum of overlapping signs and symptoms better classified as FHL dysfunction in the presence of FHL stenosing tenosynovitis or tendinosis [6]. This presentation would require a flexible approach to management in which the multimodal treatments are constantly modified based on presentation and response to treatment at each visit. The patient should not be discharged from care at a discrete point, but rather be allowed to follow up as needed until all functional goals are met. In this case, the rehabilitation period encompassed approximately eight months.

This case study has several limitations. With a single case study, making generalizations on whether a treatment approach would produce similar results with other patients is difficult. Patients with FHL dysfunction may present with signs or symptoms consistent with several different underlying pathologies. A larger case-controlled study or a prospective cohort study is warranted to better understand the prevalence of patients with similar presentations and response to treatment. However, to develop a large cohort study, the number of patients necessary to meet statistical significance may be considerable, especially when several treatment groups and multiple outcome variables are being studied.

## Conclusions

FHL dysfunction includes a cluster or of different pathologies related to medial arch pain. Patients may incur a long and extensive workup by several specialties that can be recalcitrant to treatment. Our case describes a patient with medial arch pain that underwent two rehabilitation periods with similar presentations and evolving differentials. Instead of a treatment plan directed at a discrete diagnosis, the patient experienced full resolution and no further symptoms two years after undergoing a well-rounded, multimodal rehabilitation program that adapted to the changing signs and symptoms. This case demonstrates the potential benefit of applying for an adaptable rehabilitation program as a definitive treatment option for FHL dysfunction.
